# Cascade Amplifying Electrochemical Bioanalysis for Zearalenone Detection in Agricultural Products: Utilizing a Glucose–Fenton–HQ System on Bimetallic–ZIF@CNP Nanocomposites

**DOI:** 10.3390/foods13193192

**Published:** 2024-10-08

**Authors:** Guoxing Liu, Zhaoying Liu, Yumeng Sun, Mingna Sun, Jinsheng Duan, Ye Tian, Daolin Du, Ming Li

**Affiliations:** 1 School of the Environment and Safety Engineering, Jiangsu University, Zhenjiang 212013, China; liuguoxing@stmail.ujs.edu.cn (G.L.); lucy4089@ujs.edu.cn (Z.L.); sunyumeng@stmail.ujs.edu.cn (Y.S.); tianye@stmail.ujs.edu.cn (Y.T.); 2 School of Information Security, Chongqing College of Mobile Communication, Chongqing 401420, China; 3 Key Laboratory of Agro-Product Safety Risk Evaluation, Institute of Plant Protection and Agro-Product Safety, Anhui Academy of Agricultural Sciences, Ministry of Agriculture, Hefei 230031, China; sunmingna@aaas.org.cn (M.S.); duanjinsheng@aaas.org.cn (J.D.); 4 School of Emergency Management, School of the Environment and Safety Engineering, Jingjiang College, Jiangsu University, Zhenjiang 212013, China; ddl@ujs.edu.cn; 5 School of Environmental Science and Engineering, Jiangsu Collaborative Innovation Center of Technology and Material of Water Treatment, Suzhou University of Science and Technology, Suzhou 215009, China

**Keywords:** electrochemical bioanalysis, B-ZIF@CNP, glucose–Fenton–HQ system, cascade amplification, ultrasensitive detection

## Abstract

The integration of advanced materials and signaling cascade strategies is a promising and highly relevant topic for enhancing the performance of bioanalysis. In this study, a three-stage cascade amplification electrochemical bioanalysis (TCAE-bioanalysis) was developed and evaluated for the detection of zearalenone (ZEN). This method couples immunoreaction with a glucose–Fenton–hydroquinone (HQ) system on bimetallic–ZIF (B-ZIF)@CNP nanocomposites. The B-ZIF@CNP-modified gold electrode (AuE) was prepared, offering high conductivity and an excellent reaction interface. The immunoreaction introduced glucose oxidase (GOx) into the glucose–Fenton–HQ system, generating an abundant electron signal. The method achieved an ultrasensitive limit of detection (LOD) as low as 0.87 pg/mL, with an IC_50_ value of 30.8 pg/mL, representing a 229-fold enhancement in sensitivity compared to ELISA using the same monoclonal antibody (McAb). The specificity, reliability, and practicality of this approach were thoroughly demonstrated for agricultural product samples. Additionally, the TCAE-bioanalysis offers several advantages, including simplified preparation for advanced B-ZIF@CNP, a convenient detection system, and the use of common and environmentally friendly reagents. This study presents a comprehensive approach to improving electrochemical bioanalysis and may also expand the application of signaling cascades and environmentally friendly techniques in other biosensing or diagnostic contexts.

## 1. Introduction

The field of bioanalysis has garnered significant attention for its applications in sensing detection and clinical diagnostics, primarily due to its speed, convenience, and cost-effectiveness. These advantages allow for bioanalysis to overcome some of the limitations associated with traditional detection methods [1,2]. Utilizing specific recognition elements such as antibodies or aptamers [3], bioanalysis methods predominantly include colorimetric [4,5], fluorescent [6], chemiluminescent [7], chromatographic [8], and electrochemical techniques [9,10]. Among these, electrochemical bioanalysis is particularly valued for its sensitive signal platform, high signal-to-noise (S/N) ratio, minimal sample requirements, reproducibility, and capability for real-time point-of-care monitoring of various analytes [11,12]. Moreover, electrochemical signals are advantageous in avoiding optical or fluorescent interference from complex media [13,14]. To further enhance the performance and applicability of electrochemical bioanalysis, ongoing innovations in materials and methodologies are essential.

As the core component in electrochemical bioanalysis, electrode modification plays a critical role in promoting electron transfer and immobilizing the bio-recognition layer [15,16]. Nanomaterials, widely utilized in bioanalysis, are known for their excellent physicochemical properties [17,18]. Among these, zeolitic imidazolate framework (ZIF) nanoparticles are promising candidates due to their biocompatibility, immobilization capacity, photothermal stability, and chemical robustness [19]. However, a major limitation of most ZIF nanoparticles is their insulating nature, which restricts their direct application due to poor electrocatalytic ability and low conductivity [20,21]. To address this issue, the conductivity of ZIF-67 has been enhanced by modification with gold nanoparticles, enabling the development of an electrochemical detection method for C-reactive proteins [22]. Additionally, the synergistic effects of SnO_2_, gC_3_N_4_, and ZIF-8 have been shown to impart SnO_2_@ZIF-8/gC_3_N_4_ nanocomposites with excellent catalytic activity and conductivity, achieving a limit of detection (LOD) of 0.565 μmol/L for p-nitrophenol [23]. Similarly, a hybrid material composed of XC-72 and Zn/Ni-ZIF has been employed on a glassy carbon electrode for the detection of Pb and Cu ions [24]. Consequently, nanocomposites that couple ZIF nanoparticles with electroactive materials hold significant potential for enhancing electrode conductivity and the sensitivity of bioanalysis.

Considerable efforts have been made to improve the electron signal in electrochemical bioanalysis [25,26]. In contrast to the aforementioned nanocomposites, cascade amplification aims to boost signal intensity by integrating multiple signaling strategies [27]. For example, a multiplex CRISPR/Cas12a microfluidic paper-based analytical device was developed for high-throughput analysis of the arsM gene in paddy soil, using a cascade amplification driven by crRNA-enhanced Cas12a and horseradish peroxidase (HRP)-modified probes [28]. Furthermore, a cascade signaling aptasensor for ochratoxin A (OTA) was developed by coupling cyclic digestion and metal enhancement effects [29]. Given the need for practical and environmentally friendly solutions, developing electrochemical bioanalysis methods that couple novel nanocomposites with signal cascade amplification strategies is highly desirable.

In this study, an ultrasensitive and environmentally friendly TCAE-bioanalysis is proposed by integrating bimetallic–ZIF (B-ZIF)@CNP nanocomposites, enzyme immunoreactions, a glucose–Fenton–HQ system, and electrochemical detection. The B-ZIF@CNP nanocomposites exhibited high conductivity and excellent biocompatibility. The gold electrode (AuE) was modified with B-ZIF@CNP nanocomposites and immobilized with antigen. During the immunoreaction step, the level of zearalenone (ZEN) was inversely proportional to the amount of glucose oxidase (GOx). The GOx-induced glucose–Fenton–HQ system facilitated a signaling cascade that generated abundant electron signals, enabling ultrasensitive electrochemical bioanalysis. The proposed combination of B-ZIF@CNP nanocomposites and the glucose–Fenton–HQ system is expected to improve electrochemical bioanalysis, paving the way for the creation of ultrasensitive and environmentally friendly detection methods for other analytes.

## 2. Materials and Methods

### 2.1. Reagents and Apparatus

The materials used in this study, including Zn(NO_3_)_2_·6H_2_O (99.0% purity), Ni(NO_3_)_2_·6H_2_O (99.0% purity), carbon nanopowder (CNP, C, 99.9% purity), and Al_2_O_3_ powders (99.9% purity), were provided by Tansoole Co., Ltd. (Shanghai, China). Other reagents, such as nafion (C_9_HF_17_O_5_S, 5.0 wt%), glucose monohydrate (C_6_H_12_O_6_·H_2_O, 97.5% purity), FeSO_4_·7H_2_O (99.0% purity), 2-methylimidazole (C_4_H_6_N_2_, 98.0% purity), and hydroquinone (HQ, C_6_H_6_O_2_, 98.0% purity), were supplied by Macklin Biochemical Co., Ltd. (Shanghai, China). The ZEN (C_18_H_22_O_5_, standard substance) and other standards were obtained from Pribolab Pte., Ltd. (Qingdao, China). N-hydroxysuccinimide (NHS, C_4_H_5_NO_3_, 98.0% purity) and N-(3-dimethylaminopropyl)-N-ethylcarbodiimide (EDC, C_8_H_17_N_3_, 98.0% purity) were provided by Aladdin Chemistry Co., Ltd. (Shanghai, China). The goat–anti-mouse IgG biotin conjugate (Anti-IgG-Bio), avidin-conjugated glucose oxidase (A-GOx), and bovine serum albumin (BSA) were purchased from Solarbio Co., Ltd. (Beijing, China). The antigen (Ag, ZEN-BSA) and monoclonal antibody (Ab, ZEN-McAb) were prepared in our laboratory [30], with a high affinity constant of 5.775 × 10^9^ L/mol for ZEN-McAb (Figure S1). Phosphate-buffered saline (PBS, 0.01 mol/L, pH 7.4), carbonate-buffered saline (CBS, 0.1 mol/L, pH 9.6), and the glucose–Fenton–HQ reaction solution were used in the experiments.

Nanoparticle morphology was characterized using an S-4800 scanning electron microscope (SEM) (Hitachi, Japan), and X-ray diffraction (XRD) patterns were measured on a D8 Advance A25 (Bruker, Germany). All electrochemical measurements, including cyclic voltammetry (CV), electrochemical impedance spectroscopy (EIS), and differential pulse voltammetry (DPV), were performed on a CHI660E workstation (Chenhua Instruments Co., Ltd., Shanghai, China). The LC-MS/MS method was conducted on an 8030 LC system coupled with a Waters BEH C18 column (100 mm × 2.1 mm, 1.7 μm).

### 2.2. Synthesis of B-ZIF@CNP Nanocomposites

**Figure 1:**
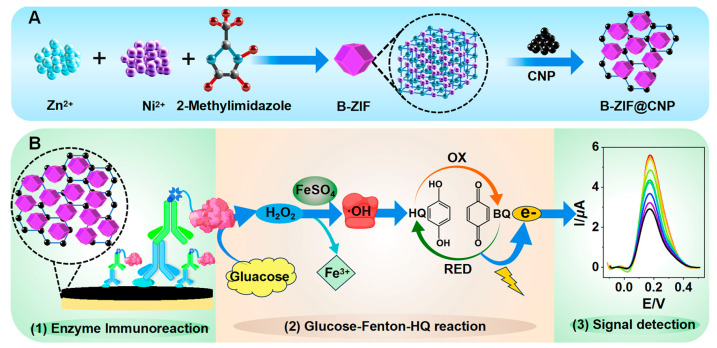
Schematic diagram of TCAE-bioanalysis. (**A**) Synthesis of B-ZIF@CNP nanocomposites; (**B**) procedure of TCAE-bioanalysis.

As illustrated in Figure 1A, the B-ZIF@CNP nanocomposites were synthesized and characterized [31].

First, 2-methylimidazole (5.296 g, 64.5 mmol), Zn(NO_3_)_2_·6H_2_O (1.188 g, 4.0 mmol), and Ni(NO_3_)_2_·6H_2_O (1.172 g, 4.0 mmol) were dissolved in methanol (100 mL) and stirred thoroughly at 25 °C for 24 h. The resulting purple precipitate was obtained by centrifugation and washed three times with methanol (10 mL per wash). After drying under vacuum at 60 °C for 12 h, the B-ZIF powder was collected. Next, the B-ZIF powder (6.0 mg), CNP (3.0 mg), and nafion (1.0 wt%, 150 µL) were added to ethanol (5.0 mL) and sonicated at 25 °C for 2 h to form the B-ZIF@CNP nanocomposites. The SEM and XRD measurements were then performed to evaluate the characteristics of B-ZIF, CNP, and B-ZIF@CNP nanocomposites.

### 2.3. Modification of Electrodes

Before use, the bare AuE (2.0 mm, working electrode) was polished with three sizes of Al_2_O_3_ powders (1.0, 0.3, and 0.05 µm). After sonication in ethanol and ultrapure water, 10 μL of the B-ZIF@CNP nanocomposite solution was added to the AuE and dried under an infrared lamp. The B-ZIF@CNP-modified AuE was then activated using the EDC/NHS method (10 μL, 0.51 mg: 0.99 mg, in CBS buffer) at 25 °C for 30 min. Subsequently, ZEN-BSA (10 μL, 6.25 μg/mL, in CBS buffer) was immobilized on the activated electrode at 4 °C overnight. Specifically, the carboxyl groups on the B-ZIF@CNP nanocomposites conjugated with hydroxyl groups on sulfo-NHS to form an intermediate product under EDC. An amide bond was then easily generated between the carboxyl groups on the B-ZIF@CNP nanocomposites and amino groups on ZEN-BSA (Figure S2). After each step, the AuE was washed to remove unbound biochemical materials. Finally, the B-ZIF@CNP/Ag-modified AuE was obtained and stored at 4 °C, enabling specific binding with ZEN-McAb through immunoreaction. The platinum (Pt) electrode (auxiliary electrode) and calomel electrode (reference electrode) were also cleaned with ultrapure water.

### 2.4. Procedure of TCAE-Bioanalysis

The three-stage cascade amplification process involved B-ZIF@CNP modification, enzyme immunoreaction, and the glucose–Fenton–HQ reaction. The TCAE-bioanalysis procedure is presented in Figure 1B, which comprises three main steps following the preparation of the B-ZIF@CNP/Ag-modified AuE:

(1) Enzyme Immunoreaction Step: The ZEN standard (or sample solution, 5.0 μL, in PBS buffer) and ZEN-McAb (5.0 μL, 2.5 μg/mL, in PBS buffer) were pre-mixed and incubated at 40 °C for 30 min. The Anti-IgG-Bio (10 μL, 2.5 μg/mL, in PBS buffer) and A-GOx (10 μL, 10 μg/mL, in PBS buffer) were also pre-incubated. The pretreated solutions were sequentially applied, and both were incubated at 40 °C for 30 min. After each incubation step, the AuE was washed with ultrapure water. Finally, the B-ZIF@CNP/Ag/Ab/Anti-IgG-Bio/A-GOx-modified AuE was used to introduce the key enzyme GOx into the detection system.
(2) Glucose–Fenton–HQ Reaction Step: The principle of the glucose–Fenton–HQ reaction is that the introduced GOx can catalyze and reduce O_2_ into H_2_O_2_ in the presence of glucose, followed by the FeSO_4_/H_2_O_2_-based Fenton reaction producing hydroxyl radicals (∙OH). The oxidation (OX) and reduction (RED) reactions between HQ and benzoquinone (BQ) are then activated by ∙OH, generating an abundant electron signal. Specifically, the Pt electrode, calomel electrode, and B-ZIF@CNP/Ag/Ab/Anti-IgG-Bio/A-GOx-modified AuE were placed in the glucose–Fenton–HQ reaction solution (3.0 mL) at 25 °C for 5 min, and the electron signal was detected using the DPV method.
(3) Signal Detection Step: The electron signal was reflected by the peak current intensity (I, μA) in the DPV curve, which was negatively correlated with the ZEN level. For the DPV curve, the response time was 120 s, and the amplitude was approximately 0.05 V, with a voltage (E, V) ranging from −0.1 V to +0.5 V.

### 2.5. Electrochemical Experiment

The electrochemical behavior was investigated using the EIS and CV methods in [Fe(CN)_6_]^3−^/^4−^ solution (5.0 mmol/L, including 0.1 mol/L KCl) following the sequential modification of the following biochemical materials: B-ZIF, B-ZIF@CNP, ZEN-BSA, ZEN-McAb, Anti-IgG-Bio, and A-GOx. The DPV curves were tested to select the optimal glucose–Fenton–HQ reaction solution by varying the ratios of glucose monohydrate, FeSO_4_·7H_2_O, and HQ. Additionally, the DPV curve of the TCAE-bioanalysis was evaluated both in the absence and presence of ZEN.

### 2.6. Establishment of TCAE-Bioanalysis

Key experimental parameters, including the ratio of B-ZIF, pH value, ZEN-BSA concentration, ZEN-McAb concentration, Anti-IgG-Bio concentration, A-GOx concentration, and competitive time, were optimized using the TCAE-bioanalysis. These parameters were evaluated based on peak current (I) values at 0.17 V. Under optimal conditions, the DPV curves for a series of ZEN standards were tested. A sigmoidal standard curve was obtained by plotting the logarithm of ZEN levels against corresponding I values using Origin 2022 software. The LOD (defined as the 10% inhibiting concentration for I value, IC_10_), the 50% inhibiting concentration (IC_50_), and the detection range (IC_10_−IC_90_) were calculated to assess the sensitivity of the TCAE-bioanalysis [32,33]. The method was further evaluated through *t*-tests comparing interday and intraday measurements, with T statistics (T) and P values (P) calculated using SPSS 26.0 software. Cross-reactivity (CR, %) values were determined by performing the TCAE-bioanalysis for ZEN and other analytes, including *α*-zearalenol (*α*-ZEL), *β*-zearalenol (*β*-ZEL), *α*-zearalanol (*α*-ZAL), *β*-zearalanol (*β*-ZAL), OTA, aflatoxin B_1_ (AFB_1_), deoxynivalenol (DON), and fumonisin B1 (FB1).

### 2.7. Application of TCAE-Bioanalysis

For ZEN-spiked samples (0, 50, 500, and 5000 pg/g), the pretreatment and extraction processes were conducted according to a previously reported method [34]. After the extracts were analyzed via TCAE-bioanalysis, the recoveries and relative standard deviation (RSD, %) values were assessed. *t*-tests were performed to compare spiked and detectable concentrations. Additionally, the proposed TCAE-bioanalysis and referenced LC-MS/MS [35,36] were used to investigate authentic samples from feed processing and market sources. The correlation of ZEN-positive results between the TCAE-bioanalysis and LC-MS/MS was also evaluated.

## 3. Results and Discussion

### 3.1. Characterization of B-ZIF@CNP Nanocomposites

**Figure 2:**
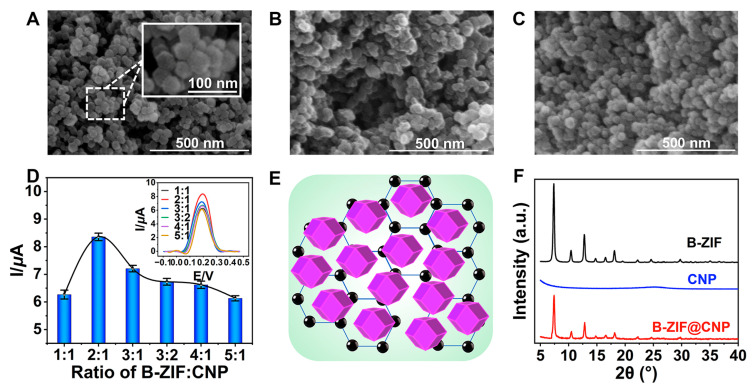
Characterizations of nanoparticles. (**A**) SEM of B-ZIF; (**B**) SEM of CNP; (**C**) SEM of B-ZIF@CNP nanocomposites; (**D**) ratio of B-ZIF and CNP for B-ZIF@CNP; (**E**) mode of B-ZIF@CNP nanocomposites; (**F**) XRD patterns of nanoparticles.

The quality of B-ZIF@CNP nanocomposites was crucial for ensuring the conductivity and reactive interface of the AuE. The morphologies of B-ZIF, CNP, and B-ZIF@CNP were characterized by SEM images. The B-ZIF nanoparticles displayed a uniform hexagonal morphology with an average diameter of approximately 40 nm (Figure 2A), while the CNP nanoparticles exhibited uniform size distribution (Figure 2B). In Figure 2C, the B-ZIF@CNP nanocomposites exhibited a rough surface and three-dimensional morphology due to the agglomeration of the two types of particles. The highest I value was observed when the B-ZIF ratio was 2:1 (Figure 2D). Using the optimal ratio of B-ZIF and CNP, uniform B-ZIF@CNP nanocomposites were prepared under the dispersion effect of nafion (Figure 2E). The composition and crystal structure of the nanoparticles were analyzed using XRD patterns (Figure 2F). For B-ZIF (black curve), diffraction peaks appeared at 2θ values of 7.2°, 10.2°, 12.4°, 14.6°, 16.6°, 18.0°, 22.0°, 24.4°, and 29.3°, corresponding to the reported planes of the cubic lattice (011, 002, 112, 022, 013, 222, 114, 233, and 004) [24]. The diffraction peaks of B-ZIF@CNP (red curve) combined the characteristics of B-ZIF and CNP, showing a weaker but similar XRD pattern. These results demonstrated that portions of the crystal planes of B-ZIF were covered by CNP, confirming the successful synthesis of B-ZIF@CNP nanocomposites.

### 3.2. Feasibility of TCAE-Bioanalysis

**Figure 3:**
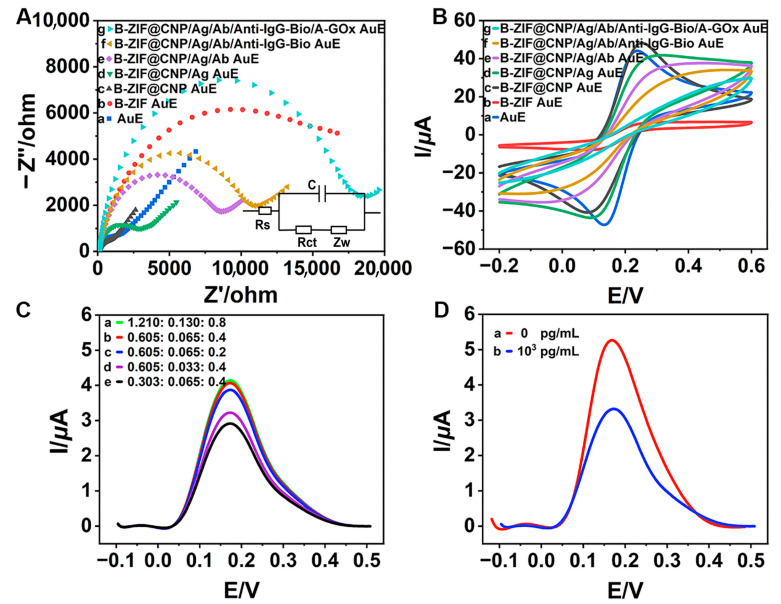
Electrochemical characterizations and feasibility studies: (**A**) EIS diagram for modified AuE (inset: equivalent circuit model); (**B**) CV diagram for modified AuE; (**C**) ratio of glucose monohydrate·7H_2_O (mmol: mmol: mmol); (**D**) feasibility of detecting ZEN.

The CV and EIS curves were analyzed to better understand the electrochemical behavior during the modification process. In the Nyquist EIS diagram shown in Figure 3A, the B-ZIF (b, red curve) exhibited a larger semicircular arc diameter compared to the bare AuE (a, blue curve), indicating that the B-ZIF increased electrical resistance, which hindered electron signal transfer. Notably, the arc diameter of the B-ZIF@CNP nanocomposites (c, black curve) was significantly smaller, even less than that of the bare AuE, suggesting that the presence of CNP enhanced the conductivity of the bare AuE and substantially improved electron signal transfer. When modified with B-ZIF, the charge transfer resistance (Rct) of the AuE increased to 11,094 Ω, indicating worse conductivity (Table S1). Conversely, the Rct value decreased to 682 Ω with the B-ZIF@CNP nanocomposites, achieving the lowest resistance and optimal conductivity. The synergistic effects between B-ZIF and CNP demonstrated superior conductivity and effective preparation for B-ZIF@CNP. Following this, the arc diameters gradually increased with the successive modifications of ZEN-BSA (d, green curve), ZEN-McAb (e, purple curve), Anti-IgG-Bio (f, yellow curve), and A-GOx (g, cyan curve) as the biochemical materials increased resistance and limited electron signal transfer.

In the CV diagram in Figure 3B, the highest current (I) value for B-ZIF@CNP (c, black curve) further confirmed its excellent conductivity, beneficial for enhancing the AuE. The progressively decreasing I values (d–g curves) also indicated successful biochemical modification on the AuE. Additionally, the values of the electrochemical parameter and electrochemical surface area (ECSA), as shown in Tables S1 and S2, further validated the excellent conductivity of B-ZIF@CNP and the successful modification with biochemical materials.

When the ratio of glucose monohydrate to FeSO_4_·7H_2_O to HQ reached the optimal value (b, red curve, Figure 3C), the DPV curve peaked, leading to excellent electron yield. The optimal glucose–Fenton–HQ reaction solution was prepared by dissolving glucose monohydrate (0.12 g, 0.605 mmol), FeSO_4_·7H_2_O (0.018 g, 0.065 mmol), and HQ (0.044 g, 0.4 mmol) into PBS buffer (40 mL, pH 7.4). Moreover, the DPV curve significantly decreased after adding ZEN (1.0 × 10^3^ pg/mL, b, red curve) compared to the absence of ZEN (0 pg/mL, a, blue curve), verifying the feasibility of TCAE-bioanalysis for detecting ZEN (Figure 3D).

### 3.3. Optimization

**Figure 4:**
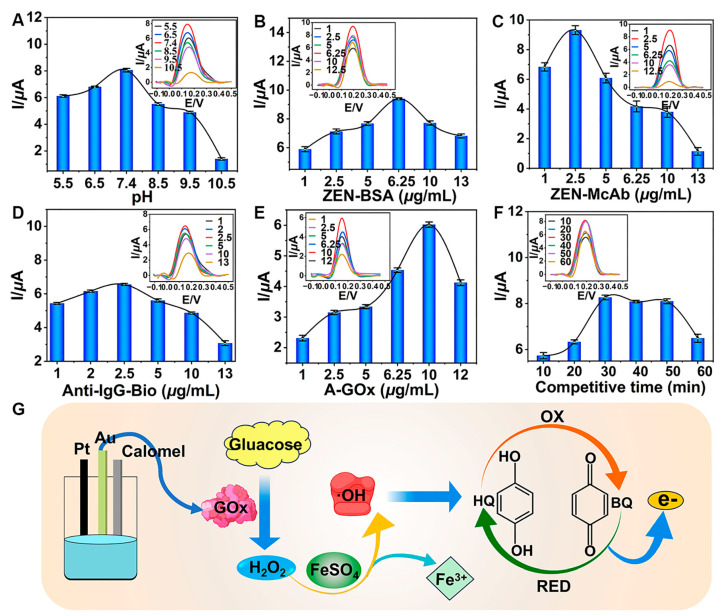
Optimization of key parameters: (**A**) pH; (**B**) ZEN-BSA; (**C**) ZEN-McAb; (**D**) Anti-IgG-Bio; (**E**) A-GOx; (**F**) competitive time; (**G**) schematic for generating electron signal.

Optimizing key parameters was crucial for the TCAE-bioanalysis. The I values increased as the pH rose from 5.5 to 7.4 (Figure 4A). However, when the pH exceeded 7.4, the I values began to decline, indicating that pH 7.4 was optimal for detection. This was due to the fact that the activity of immunoreaction between ZEN-BSA and ZEN-McAb might be gradually inhibited by the higher pH values, resulting in the lessened introduction of GOx. As shown in Figure 4B, the I values of the DPV curve displayed a wavelike trend, reaching a maximum at a ZEN-BSA concentration of 6.25 μg/mL. Similarly, the optimized concentrations for ZEN-McAb, Anti-IgG-Bio, and A-GOx were determined to be 2.5 μg/mL (Figure 4C), 2.5 μg/mL (Figure 4D), and 10 μg/mL (Figure 4E), respectively. The surface concentration of ZEN-McAb on AuE was 0.796 μg/mm². The I values plateaued between 30 and 50 min and then decreased, indicating that longer competition times were unfavorable (Figure 4F). The final optimal parameters were pH 7.4, 6.25 μg/mL ZEN-BSA, 2.5 μg/mL ZEN-McAb, 2.5 μg/mL Anti-IgG-Bio, 10 μg/mL A-GOx, and a 30 min competitive time. Under these optimal conditions and during the immunoreaction step, GOx was introduced into the glucose–Fenton–HQ system to generate a substantial electron signal (Figure 4G).

### 3.4. Sensitivity

**Figure 5:**
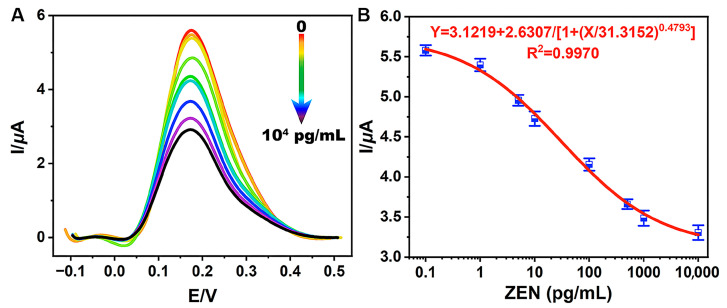
Electrical signals of DPV for series of ZEN standards (**A**) and the standard curve (**B**) for TCAE-bioanalysis.

The sensitivity of TCAE-bioanalysis was assessed by detecting a series of ZEN standards. When ZEN was absent (0 pg/mL), the biochemical materials (such as ZEN-McAb, Anti-IgG-Bio, and A-GOx) could adsorb onto the B-ZIF@CNP/Ag-modified AuE as much as possible. In this scenario, the glucose–Fenton–HQ system was maximally activated, resulting in the highest DPV curve and I value (Figure 5A). Conversely, as the ZEN concentration increased from 0.1 pg/mL to 1.0 × 10^4^ pg/mL, the amount of biochemical material on the AuE gradually decreased, leading to a corresponding decrease in enzymatic activity and the DPV curve.

As shown in Figure 5B, ZEN levels (X) and I values (Y) were inversely proportional. The sigmoidal standard curve of TCAE-bioanalysis was obtained using the four-parameter equation Y = 3.1219 + 2.6307/[1 + (X/31.3152) 0.4793] (R^2^ = 0.9970). The LOD and IC_50_ values were defined as 0.87 pg/mL and 30.8 pg/mL, respectively, with a detection range of 0.87–1058.5 pg/mL. With an LOD at the pg/mL level and significantly below the maximum limit values for ZEN [37,38], the TCAE-bioanalysis method meets the demand for the ultrasensitive detection of ZEN. The sensitivity of TCAE-bioanalysis has been significantly enhanced by the novel nanocomposites and three-stage cascade amplification strategies. The *t*-test results between intraday and interday measurements showed no significant differences at a 95% confidence interval (Table S3), demonstrating the reliability of the proposed detection method.

### 3.5. Specificity

**Figure 6:**
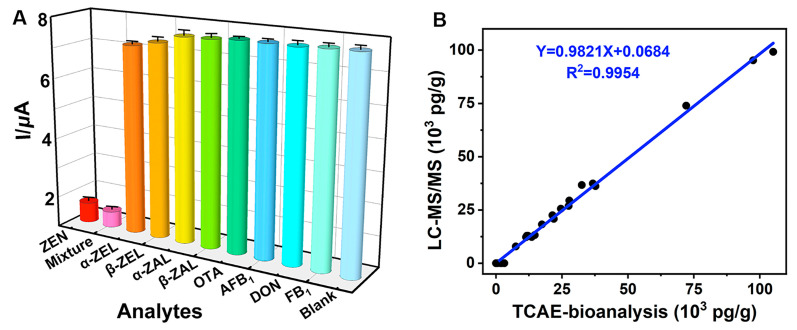
Specificity of TCAE-bioanalysis (**A**) and correlation for ZEN-positive samples (**B**).

The CR test was used to evaluate the specificity of TCAE-bioanalysis (Figure 6A). The results showed that ZEN (1.0 × 10^3^ pg/mL) and a mixture (ZEN and other analytes, 1.0 × 10^3^ pg/mL) had the lowest I values, while other analytes (1.0 × 10^3^ pg/mL) and the blank (0 pg/mL) had the highest I values. These results indicated that other analytes had minimal impact on electron signal inhibition, while GOx was maximally immobilized on the AuE. Specifically, the mixture had the highest CR value at 108.4%, with CR values of 7.31% and 5.07% for *α*-ZEL and *β*-ZEL, respectively (Table S4). CR values for other analytes were negligible (CR < 1.55%). The cumulative effects of ZEN and its analogs (primarily from *α*-ZEL and *β*-ZEL) resulted in the highest CR value for the mixture. Overall, the TCAE-bioanalysis demonstrated ideal specificity for ZEN.

### 3.6. Detection of ZEN-Contaminated Samples

The accuracy and precision of TCAE-bioanalysis were evaluated by assessing recovery and RSD values for ZEN-spiked samples (including wheat, peanuts, and feed). Overall, recoveries ranged from 76.4% to 108.2%, with RSD values ranging from 5.2% to 10.2% (Table S5). Moreover, the *t*-test results (T = 0.041, *p* = 0.967 > 0.05) indicated no significant differences between the spiked and detected concentrations at a 95% confidence interval. The proposed TCAE-bioanalysis is a feasible and promising strategy for detecting ZEN contamination at the pg/mL level.

Additionally, a total of 26 authentic samples, highly suspected of ZEN contamination, were assessed using TCAE-bioanalysis and verified by LC-MS/MS (Table 1). For the TCAE-bioanalysis, 24 authentic samples showed ZEN-positive results ranging from 56.3 pg/g to 105.1 × 10^3^ pg/g, with standard deviation (SD) values from 4.38 pg/g to 5.12 × 10^3^ pg/g. In contrast, ZEN-positive levels detected by LC-MS/MS in 18 authentic samples ranged from 7.93 × 10^3^ pg/g to 99.2 × 10^3^ pg/g, with SD values from 0.53 × 10^3^ pg/g to 7.33 × 10^3^ pg/g. Notably, six ZEN-positive samples detected by TCAE-bioanalysis (56.3 pg/g to 3.15 × 10^3^ pg/g) were judged as ZEN-negative by LC-MS/MS due to its higher LOD value (7.4 × 10^3^ pg/g). The consistent ZEN-positive results between TCAE-bioanalysis and LC-MS/MS were represented by the equation Y = 0.9821X + 0.0684 (R^2^ = 0.9954) (Figure 6B), further demonstrating the reliability and practicality of TCAE-bioanalysis.

### 3.7. Comparison with Other Bioanalysis Methods

By utilizing advanced B-ZIF@CNP to modify AuE and an efficient glucose–Fenton–HQ system to generate electron signals, this integrated research has significantly enhanced the performance of electrochemical bioanalysis. The proposed TCAE-bioanalysis employs a three-stage cascade amplification system, unlike most reported methods, which typically use a single amplification strategy. As shown in Table 2, the sensitivity of TCAE-bioanalysis was significantly improved to the pg/mL level, showing a 229-fold and 574-fold enhancement compared to conventional ELISA [30] and ICA [37] using the same ZEN-McAb. Compared to other optical and electrochemical bioanalysis methods [4,6,18,26], TCAE-bioanalysis also demonstrated superior sensitivity. Many reported detection strategies of ZEN for food safety face challenges such as complex preparation, weak signal intensity, and the use of toxic and expensive reagents [39]. In contrast, TCAE-bioanalysis offers several advantages, including a simple preparation process for B-ZIF@CNP, excellent conductivity of the B-ZIF@CNP-modified AuE, an outstanding glucose–Fenton–HQ system for generating electron signals, and the use of common and environmentally friendly reagents. Additionally, the glucose–Fenton–HQ reaction solution combined enzyme catalysis, OH generation, and electron signal transfer functions within a single system. With pretreatment and prefabrication, the TCAE-bioanalysis requires only 1 h for the enzyme immunoreaction step and 5 min for the glucose–Fenton–HQ reaction, simplifying and accelerating the detection procedure. More effort can be put into improving the standardized application and portable electrochemical workstation for this TCAE-bioanalysis.

## 4. Conclusions

In summary, a novel three-stage cascade signal amplification strategy based on B-ZIF@CNP-modified AuE, GOx-coupled immunoreaction, and glucose–Fenton–HQ-induced electron signal generation has been proposed and applied to develop TCAE-bioanalysis for detecting ZEN. The B-ZIF@CNP nanocomposites were synthesized using a straightforward and environmentally friendly procedure, providing high conductivity and an excellent reactive interface. The glucose–Fenton–HQ system integrated multistep signal conversion into a single reaction solution to generate abundant electron signals, resulting in simplified and eco-friendly detection. With an LOD of 0.87 pg/mL, this method is 229-fold more sensitive than conventional ELISA, making it a viable alternative for monitoring ZEN at the pg/mL level. The specificity, reliability, and practicality of TCAE-bioanalysis have been demonstrated to be satisfactory. This study not only provides a potential application for B-ZIF@CNP-modified AuE and the glucose–Fenton–HQ system but also broadens the application of multistage cascade signal amplification for ultrasensitive and environmentally friendly detection of other analytes. It is worth promoting the standardized application and improving the portable electrochemical workstation in the future, which might be more beneficial to enhance their application in bioanalysis and diagnosis.

## Figures and Tables

**Table 1:** ZEN-positive authentic samples by TCAE-bioanalysis and LC-MS/MS.

Sample	TCAE-Bioanalysis ^a^ (Mean ± SD, pg/g)	LC-MS/MS (Mean ± SD, pg/g)	Sample	TCAE-Bioanalysis (Mean ± SD, pg/g)	LC-MS/MS (Mean ± SD, pg/g)
Corn-1	(17.4 ± 1.23) × 10^3^	(18.2 ± 0.96) × 10^3^	Feed-1	ND ^b^	ND ^c^
Corn-2	(14.7 ± 0.61) × 10^3^	(13.2 ± 0.57) × 10^3^	Feed-2	(36.7 ± 2.41) × 10^3^	(37.4 ± 1.85) × 10^3^
Corn-3	(13.5 ±0.86) × 10^3^	(12.3 ± 0.85) × 10^3^	Feed-3	(3.15± 0.33) × 10^3^	ND ^c^
Corn-4	(27.8 ± 1.74) × 10^3^	(29.4 ± 2.40) × 10^3^	Feed-4	(21.9 ± 2.25) × 10^3^	(20.8 ± 2.70) × 10^3^
Corn-5	(105.1 ± 5.12) × 10^3^	(99.2 ± 4.30) × 10^3^	Feed-5	(7.50 ± 0.89) × 10^3^	(7.93 ± 0.53) × 10^3^
Corn-6	(97.5 ± 4.01) × 10^3^	(95.2 ± 7.33) × 10^3^	Feed-6	(27.5 ± 2.14) × 10^3^	(26.9 ± 1.99) × 10^3^
Peanut-1	(11.4 ± 0.55) × 10^3^	(12.2 ± 0.63) × 10^3^	Wheat-1	(1.76± 0.17) × 10^3^	ND ^c^
Peanut-2	528.1 ± 30.1	ND ^c^	Wheat-2	(24.6 ± 2.10) × 10^3^	(25.6 ± 1.10) × 10^3^
Peanut-3	(21.4 ± 1.81) × 10^3^	(22.5 ± 1.50) × 10^3^	Wheat-3	ND ^b^	ND ^c^
Peanut-4	(32.5 ± 2.52) × 10^3^	(36.7 ± 2.44) × 10^3^	Wheat-4	(37.7 ± 2.58) × 10^3^	(36.2 ± 3.52) × 10^3^
Peanut-5	(1.76 ± 0.12) × 10^3^	ND ^c^	Wheat-5	(2.08± 0.19) × 10^3^	ND ^c^
Peanut-6	(11.7 ± 0.42) × 10^3^	(12.9± 0.77) × 10^3^	Wheat-6	(12.1 ± 0.96) × 10^3^	(12.9 ± 0.88) × 10^3^
Peanut-7	56.3 ± 4.38	ND ^c^	Wheat-7	(72.1 ± 3.50) × 10^3^	(73.9 ± 4.77) × 10^3^

^a^ Three replicates were performed, and extracts performed 20-fold dilution. ^b^ ND: not detected, less than 17.4 pg/g of LOD for TCAE-bioanalysis. ^c^ ND: not detected, less than 7.4 × 10^3^ pg/g of LOD for LC-MS/MS.

**Table 2:** Comparison with other bioanalysis methods for ZEN.

Method	Signal	Recognition Group	Label	Detection Range (pg/mL)	LOD (pg/mL)	Reference
ELISA	Colorimetry	McAb	HRP	200–5400	200	[30]
ELASA	Colorimetry	Aptamer	HRP	380–28 × 10^3^	377	[4]
ICA	Red color	McAb	AuNP	800−40 × 10^3^	690	[8]
ICA	Red color	McAb	AuNP	500–3000	500	[37]
FIA	Fluorescence	McAb	ALP@PPI/Cu^2+^	(3.5–17.8) × 10^3^	14	[6]
SERS	Raman	McAb	MSN@AuNP	300–200 × 10^3^	6.4	[17]
EC	Electricity	Aptamer	Label-free	10−1000 × 10^3^	17	[10]
EC	Electricity	McAb	ALP	250−250 × 10^3^	250	[11]
EC	Electricity	MIP	Nanoribbon @AuNP	(1.0−500) × 10^5^	340	[26]
Dual-signal IA	Colorimetry/Electricity	McAb	ALP	200−800/125−500	40/80	[18]
TCAE-bioanalysis	Electricity	McAb	B-ZIF@CNP/glucose–Fenton–HQ	0.87−1058.5	0.87	This study

ELISA: enzyme-linked immunosorbent assay; ELASA: enzyme-linked aptamer sorbent assay; ICA: immunochromatography; FIA: fluorescence immunoassay; SERS: surface-enhanced Raman scatting; EC: electrochemistry; IA: immunoassay; MIP: molecularly imprinted; AuNPs: gold nanoparticles; ALP: alkaline phosphatase; PPI: pyrophosphate; MSNs: mesoporous silica nanoparticles.

## Data Availability

The original contributions presented in this study are included in the article/Supplementary Materials; further inquiries can be directed to the corresponding author.

## Supplementary Materials

The following supporting information can be downloaded at: https://www.mdpi.com/article/10.3390/foods13193192/s1, Figure S1: Affinity constant for ZEN-McAb; Figure S2: Chemical reaction between B-ZIF@CNP and ZEN-BSA/ZEN-McAb; Table S1: Electrochemical parameters for different modified electrodes; Table S2. Electrochemical surface area (ECSA) for each electrode; Table S3: *t*-test results for intraday and interday measurements; Table S4: Cross-reactivity of TCAE-bioanalysis for other analytes; Table S5: Recovery of TCAE-bioanalysis in spiked sample.
