# Supplementary material for: Cascade Amplifying Electrochemical Bioanalysis for Zearalenone Detection in Agricultural Products: Utilizing a Glucose–Fenton–HQ System on Bimetallic–ZIF@CNP Nanocomposites
Source: Foods. 2024 Oct 8;13(19):3192. doi: 10.3390/foods13193192 (PMC11475201; doi:10.3390/foods13193192)
Supplement: Supplementary file 1 [file foods-13-03192-s001.zip › foods-3212299-supplementary.pdf]

Supplementary materials

Figure S1. Affinity constant for ZEN-McAb.

Figure S2. Chemical reaction between B-ZIF@CNP and ZEN-BSA/ZEN-McAb.

Table S1. Electrochemical parameters for different modified electrodes.

Table S2. Electrochemical surface area (ECSA) for each electrodes.

Table S3. *t*-test results for intraday and interday measurements.

Table S4. Cross-reactivity of TCAE-bioanalysis for other analytes.

Table S5. Recovery of TCAE-bioanalysis in spiked sample.

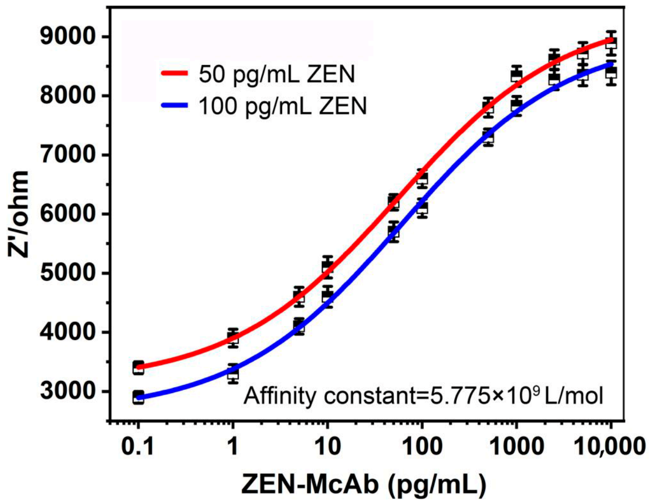

Figure S1. Affinity constant for ZEN-McAb.

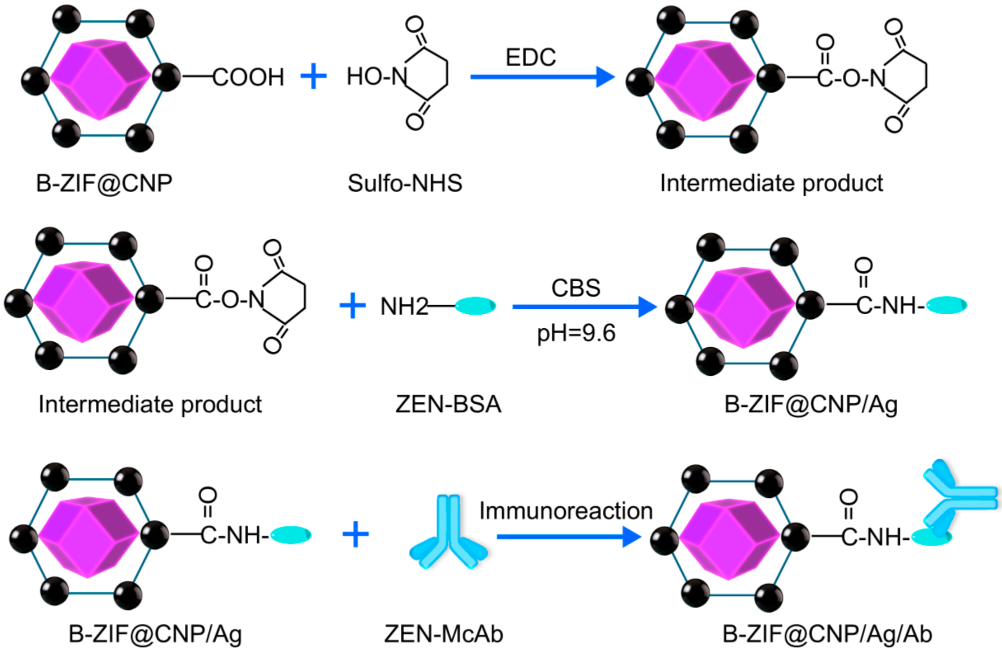

Figure S2. Chemical reaction between B-ZIF@CNP and ZEN-BSA/ZEN-McAb.

Table S1. Electrochemical parameters for different modified electrodes.

Modified electrode	Rct (Ω)	Capacitive (C, μF)	i_0 ($\times 10^{-6}$ A/cm^2)	k_0 ($\times 10^{-5}$ cm/s)
a	1279	5.2488	19.68301	65.17181
b	11094	5.2488	2.269932	7.515403
c	682	5.4375	328.9416	1089.609
d	1914	5.2053	11.71476	38.80112
e	3199	5.6326	7.006882	23.19736
f	8701	5.1557	2.576657	8.538633
g	15486	5.1364	1.448083	4.797251
Attachment	Zview software	$I_c = C \times \frac{dV}{dt}$	$i_0 = \frac{RT}{nFRct}$	$k_0 = \frac{i_0}{nFAC}$

Table S2. Electrochemical surface area (ECSA) for each electrodes.

Modified electrode	Bare AuE	B-ZIF@CNP	B-ZIF@CNP /Ag	B-ZIF@CNP /Ag/Ab	B-ZIF@CNP /Ag/Ab/Anti-IgG-GOx
ECSA (cm^2)	0.0706	0.1056	0.0637	0.0549	0.0390

Table S3. *t*-test results for intraday and interday measurements.

	ZEN (pg/mL)	T value	P value
	1.0	0.272	0.799
	5.0	1.055	0.351
Intraday	10	1.651	0.174
	100	1.452	0.220
	1000	2.025	0.113
	1.0	-0.232	0.828
	5.0	0.943	0.399
Interday	10	1.612	0.182
	100	1.433	0.225
	1000	1.373	0.242

Table S4. Cross-reactivity of TCAE-bioanalysis for other analytes.

Analyte	IC_{50} (pg/mL)	CR (%)
ZEN	30.8	100
Mixture	28.4	108.4
α-ZEL	421.3	7.31
β-ZEL	607.5	5.07
α-ZAL	1987.1	1.55
β-ZAL	2248.2	1.37
OTA	> 5000	< 0.62
AFB_1	4738.5	0.65
DON	4338.0	0.71
FB1	> 10,000	< 0.31
Blank	> 10,000	< 0.31

Table S5. Recovery of TCAE-bioanalysis in spiked samples.

Sample	Spiked (pg/g)	Found [a] (pg/g)	Recovery (%)	RSD (%)
Wheat	0	ND [b]	/	/
	50	54.1	108.2	7.6
	500	521.0	104.2	10.2
	5000	4870.5	97.4	6.9
Peanut	0	ND	/	/
	50	48.6	97.2	5.2
	500	516.5	103.3	9.3
	5000	4925.3	98.5	6.7
Feed	0	ND	/	/
	50	38.2	76.4	8.4
	500	514.0	102.8	8.9
	5000	4725.9	94.5	7.3
Attachment		T value=0.041	P value=0.967	

[a] Three replicates were performed, and extracts were performed 20-fold dilution.

[b] ND: not detected, less than 17.4 pg/g of LOD for TCAE-bioanalysis.
